# Glycogenic hepatopathy in a primitive teleost fish model: the inductive effect of high carbohydrate diet and the alleviating role of betaine

**DOI:** 10.1007/s42995-025-00301-0

**Published:** 2025-05-12

**Authors:** Jiahong Zou, Zhenwei Chen, Feifei Zheng, Xiaojuan Cao, Yuhua Zhao, Stephane Panserat, Jian Gao, Qingchao Wang

**Affiliations:** 1 College of Fisheries, Huazhong Agricultural University, Wuhan, 430070 China; 2 UMR1419 Nutrition Métabolisme Et Aquaculture, Université de Pau Et Des Pays de L’Adour, E2S UPPA, INRAE, F-64310 Saint-Pée-Sur-Nivelle, France

**Keywords:** Liver disease, Glycogenic hepatopathy, High carbohydrate, Betaine, Metabolomic

## Abstract

Carbohydrate-induced glycogenic hepatopathy is underdiagnosed and difficult to discriminate from hepatic steatosis in humans. The present study used liver biopsy and other diagnostic tools, and adopted metabolomic technology to identify glycogenic hepatopathy rather than hepatic steatosis in largemouth bass induced by high carbohydrate diet (HC). HC induced obvious liver injury in largemouth bass with disarranged hepatocytes, partial cell membrane damage, and irregular distribution of nuclei, accompanied by increased serum ALT and AST activities. PAS staining and glycogen content analysis detected significant increases in liver glycogen content, whereas intrahepatic triglyceride content and programmed cell death were not affected. Metabolomic analysis indicated that up-regulated metabolites were mainly enriched in glycogen synthesis precursors, whereas betaine and unsaturated fatty acids were down-regulated, confirming that HC induced glycogenic hepatopathy rather than hepatic steatosis in largemouth bass. The significantly decreased betaine in both serum and liver of largemouth bass served as a potential key regulator in such disease, whose supplementation could alleviate HC-induced liver injury and serum ALT activity. Betaine supplementation increased betaine and carnitine contents in liver and serum, resulting in decreased hepatic glycogen deposition and cortisol content in largemouth bass. Further study showed that dietary betaine significantly improved the capacity of largemouth bass to resist against ammonia stress. Therefore, our study established a glycogenic hepatopathy-inducing and evaluation model in a primitive teleost and also identified the betaine as the key metabolic marker and prevent strategy, which help advance the understanding of human liver diseases.

**Supplementary Information:**

The online version contains supplementary material available at 10.1007/s42995-025-00301-0.

## Introduction

Liver disease is a significant health challenge worldwide, including in China, involving primarily alcoholic and non-alcoholic fatty liver disease (NAFLD) and viral hepatitis. These have been reported to affect approximately 300 million people, worldwide. NAFLD incidence is continuously increasing, and epidemiological evidence has indicated the contributing roles of excess carbohydrate consumption in metabolic diseases including type 2 diabetes (T2DM) and NAFLD via increasing lipogenesis (Le et al. 2023). Besides lipogenesis, excess carbohydrate intake promotes glycogenesis, and the resulting accumulation of glycogen within the hepatocytes, i.e., glycogenic hepatopathy, which is usually seen in patients with poorly controlled T1DM and also those with poorly controlled T2DM (Khoury et al. 2018). Although the effects of hepatic steatosis on metabolic disease have been studied comprehensively, little information is known about the contribution of glycogenic hepatopathy in T2DM. Glycogenic hepatopathy is difficult to separate from hepatic steatosis. Thus, its prevalence is predicted to be much higher than reported. Compared to mammals, the ability of fish to utilize carbohydrates for energy supply is relatively lower (Enes et al. 2009). These are known also as the congenital diabetic patients, as they usually experience postprandial hyperglycemia for a long period with carbohydrate-rich feed intake. The elevated blood glucose promotes hepatic glucose intake, which leads to excessive glycogen or lipids accumulation in hepatocytes, thus impairing liver function (Prisingkorn et al. 2017). However, recent studies identified a novel liver disease in largemouth bass (*Micropterus salmoides*) due to a high carbohydrate diet, in which a mass of glycogen-like granules were detected in hepatocytes with vacuoles that were different from fatty liver disease (Huang et al. 2022; Romano et al. 2022). Conversely, contradictory results have been reported with increased hepatic triglyceride (TG) and total cholesterol contents induced by high carbohydrate diet (Wu et al. 2022). It would be meaningful to illustrate the effects of glycogenic hepatopathy on liver health with largemouth bass as the research model, involving development of glycogenic hepatopathy rather than hepatic steatosis under high carbohydrate intake.

Liver biopsy with the significant accumulated glycogen in hepatocytes is the gold standard for diagnosis. However, the elevated liver enzymes in routine blood tests may also diagnose glycogenic hepatopathy, as it usually regresses after tight glycemic control. The most commonly used serum tests involve aspartate transaminase (AST) and alanine aminotransferase (ALT). However, in fish, the unified diagnostic criteria of glycogenic hepatopathy are difficult as the hepatic glycogen content among different fish species varied to a large extent. Recently, high-throughput metabolomics have been used widely in identifying and quantifying multiple biomarkers simultaneously in the development and progression of diseases (Jin and Ma 2021). It would be useful to detect metabolites related to lipogenesis and glycogenesis, which could help establish detection criteria for glycogenic hepatopathy. Considering the significant influences of excess carbohydrate on liver health, searching for methods in the treatment and prevention of metabolic diseases is important. In humans, lifestyle interventions are suggested but they exhibited limited effectiveness, high costs, and great effort. Currently, there are no FDA-approved pharmacological therapies for NAFLD, but many agents that are currently prescribed for hyperglycemia have been reported to exhibit positive effects on non-alcoholic steatohepatitis (NASH) biomarkers (Xu et al. 2014). High-throughput metabolomics not only functions in identifying and quantifying multiple biomarkers simultaneously in the development and progression of diseases but also facilitates their prevention and treatment.

In fish, dietary inclusion of no-protein energy sources, including carbohydrate, minimizes the use of high-priced protein sources and leads to good floating properties for the preferred extruded feeds. Adequate inclusion of carbohydrate exhibited the protein-saving effects without adverse effects on fish growth in some fish species, whereas excessive intake of carbohydrates significantly inhibited fish growth performance via reducing feed intake and impairing the utilization of other dietary nutrients (Mohanta et al. 2010). In blunt snout bream (*Megalobrama amblycephala*), excess dietary starch resulted in increased blood glucose concentrations, hepatic insulin resistance, excess accumulated glycogen or lipids in hepatocytes, and impaired liver function (Prisingkorn et al. 2017). Largemouth bass is a carnivorous fish species of great economic value, and has been reared widely in many countries (Wang et al. 2022). In China, liver health received extensive attention, and could be influenced significantly by dietary carbohydrate content (Huang et al. 2022). Recent studies indicated the effects of high carbohydrate on the glycogenic hepatopathy rather than hepatic steatosis in largemouth bass. If correct, this would serve as an excellent research model to illustrate the contribution of glycogenic hepatopathy to hepatic disease in both human and fish with excess glucose intake. Thus, we conducted a metabolomic study along with liver biopsy and transaminase production to confirm the hypothesis, and also search for the key intermediary metabolite for the prevention and treatment of glycogenic hepatopathy in largemouth bass with excess carbohydrate intake. This is the first study to confirm carbohydrate diet-induced glycogenic hepatopathy rather than hepatic steatosis via a metabolomic study. Also, this serves as a preventative method to alleviate excess carbohydrate-induced liver damage.

## Materials and methods

### Fish feed and rearing trial

Largemouth bass (~ 48 g) used in the present study were bought from Yichang fishery farm (Hubei, China). All fish were reared in a recycling aquaculture system for two weeks in the aquaria of Huazhong Agricultural University. Water flow was maintained at 0.5 L/min, water temperature and pH were kept at 26 ± 2 °C & 7.6 ± 0.2, and water oxygen content was achieved at 85% saturation. Daily rhythm followed natural changes.

In Experiment #1, two diets with different carbohydrate levels including 12% (control diet; Con) and 20% (high carbohydrate diet; HC) were formulated (Table 1). Six tanks were arranged randomly into two groups, with 18 fish in each tank for an eight weeks feeding trial. Fish were fed with experimental diets twice daily. After eight weeks of feeding, serum, liver, and gill of five largemouth bass were sampled at three hours after the final meal for further analyses.

**Table 1:** Feed formulations in the present study

Ingredients	Con (%)	HC (%)	HC + Bet (%)
Fish meal	35	35	35
Krill meal	3	3	3
Gluten meal	4	4	4
Blood meal	2	2	2
Corn gluten meal	5	5	5
Cottonseed protein concentrate	15	15	15
Beer yeast	2.5	2.5	2.5
Wheat meal	6	10	10
Tapioca	6	10	10
Bentonite	10.3	2.3	1.3
Fish oil	5	5	5
Soy lecithin	2	2	2
Multidimensional and multi mineral premix	2	2	2
Taurine	0.5	0.5	0.5
Betaine	0	0	1
Choline chloride	0.25	0.25	0.25
Monocalcium phosphate	0.3	0.3	0.3
Calcium propionate	0.1	0.1	0.1
Ethoxyquin	0.05	0.05	0.05
Alginate	1	1	1
Proximate analysis			
Crude Protein	47.35	47.80	47.75
Crude Lipid	10.86	11.04	11.24

In Experiment #2, HC diet and another diet with 1% betaine supplementation to high carbohydrate diet (HC + Bet) were used for another eight weeks feeding trial, with all other feed formulations similar to Experiment #1. Fish in two groups were weighed for calculating weight gain rate (WGR, %) and specific growth rate (SGR, %/day) after the feeding trial continued for two, five, and eight weeks. Similar procedures were used for the eight-week feeding trial, and serum and tissue collection.

After sampling at eight weeks, the remaining 49 fish in each group were used for an ammonia stress experiment. Firstly, an ammonia stock solution was prepared with ammonium chloride (NH_4_Cl) (Merck and Company Inc, Whitehouse Station, NJ, USA). According to a preliminary experiment (Zou et al. 2023), total ammonia nitrogen (TAN) used in the present study was 13.0 ± 0.5 mg/L. Then, samples were taken for the same procedure after ammonia stress for three and seven days, respectively; five strips were taken from each group.

### Preparation of tissue sections and staining

Partial liver and gill tissues were fixed in 4% paraformaldehyde for 24 h, and then removed for alcohol gradient dehydration, cleared in xylene, dipped in wax, and embedded. The embedded tissues were sectioned at 5 μm thickness, and used for hematoxylin–eosin (H&E) staining and PAS staining, respectively. For PAS staining, the paraffin sections of liver tissue were stained with glycogen PAS solution (Periodic Acid-Schiff stain) (G1280, Solarbio, China). Frozen sections (10 μm) of liver tissue were obtained using a freezing microtome (Leica CM3050 S) and then processed using the modified oil Red O staining kit (C0158S, Beyotime, China). All the stained slides were then observed and photographed using an Olympus light microscope.

### Biochemistry assay and analysis of enzyme activities

The triglyceride (TG), total cholesterol (T-CHO), and low-density lipoprotein cholesterol (LDL-C) levels in liver tissues were evaluated using the corresponding commercial kits from Nanjing Jiancheng Bioengineering Institute (A110-1–1, A111-1–1, A113-1–1, China). Additionally, glycogen content in liver was detected with the commercial kit (BC0345, Solarbio, China).

Serum ALT and AST enzyme activities were determined and analyzed using a commercial kit from Solarbio (BC1555 & BC1565, China). Meanwhile, the apoptotic enzyme activities in the liver, including caspase-1, caspase-3, caspase-8, and caspase-9, were determined according to the manufacturer's instructions (C1101, C1115, C1151, C1157, Beyotime, China).

### TUNEL assay in liver and immunofluorescence analysis in gill

The one-step terminal deoxynucleotidyl transferase dUTP nick-end labeling (TUNEL) assay kit from Beyotime (C1086, China) was used to detect apoptotic cells in fish liver. Images were captured and analyzed using a CCD camera (Olympus, Japan). On the other hand, frozen slices of gill tissue were fixed with paraformaldehyde for 3 min and then sealed with sealing solution for 15 min for immunofluorescence assay. Anti-5-HT-1B antibody (dilution ratio 1:500, 3,112,009, Sigma) and 488 Goat anti-Rabbit IgG (dilution ratio of 1:600, AS053, Abclonal) were used as the primary and second antibody in the assay for fluorescence microscope development and photography.

### RNA extraction and qRT-PCR analysis

Total RNA from fish liver and gill tissues was extracted using TRIZOL (Invitrogen). The concentration, purity, and integrity of RNA were determined using an ultra-micro spectrophotometer (Nanodrop-2000, Germany), and 1% agarose gel electrophoresis, respectively. Afterward, 1 μg mRNA from each sample was immediately reversed after DNase treatment using a commercial kit (15662ES08, YEASEN, China). Then, qRT-PCR was performed in a Jena qTOWER^3G^ system with the diluted reversed cDNA (200 ng/μL) samples as the templates. qPCR SYBR Green Master Mix (11201ES08, YEASEN, China) was used as the qRT-PCR assay kit. *18S* was adopted as the reference gene as its expression showed no significant changes among all experimental groups. Then the relative expression level of each target gene in different groups were analyzed using the 2^−ΔΔCt^ method against *18S*. All primers used in this study are listed in Supplementary Table S1.

### High-throughput targeted metabolomics analyses in largemouth bass liver and serum

The extraction of metabolites from largemouth bass liver samples was performed as described by Gong et al. (2024). For Liquid Chromatography-Mass Spectrometry (LC–MS) analysis, these extracted liver metabolites were re-dissolved in an acetonitrile/water (1:1, v/v) solvent and centrifuged again at 14,000 *g* for 15 min at 4 ℃. The supernatants were separated by UHPLC, using an Agilent 1290 Infinity LC column, and tested by 6500 + QTRAP system (AB SCIEX), following the provided instructions of Applied Protein Technology Co., Ltd (China). Peak areas and insets of each substance were obtained by peak extraction from raw MRM data using MultiQuant or Analyst software.

Largemouth bass serum was used for targeted metabolomics assay to determine betaine, carnitine, TMA, TMAO, choline, and creatinine contents as described by Chen et al. (2023). Similarly, the betaine-related metabolites analyses were entrusted to Applied Protein Technology Co., Ltd (China), with UHPLC system, 5500 QTRAP mass spectrometer (AB SCIEX) and MultiQuant software adopted.

### Statistical analysis of data

GraphPad Prism 6 was adopted to conduct the statistical analysis of all experimental data. Differences between two samples were analyzed using Student's t-test, and the significance of difference between two groups was labeled by * (*P* < 0.05), ** (*P* < 0.01), and ***(*P* < 0.001). Cell size was measured with Image J. Data of gene expression and histological indexes in ammonia challenge experiment were subjected to one-way analysis of variances (ANOVA), followed by Tukey’ s multiple range tests at the *P* < 0.05 threshold to inspect differences among all the data. Data are representative of at least three independent replicates (mean ± SEM).

## Results

### High carbohydrate diet induced liver injury with increased intrahepatic glycogen but not lipid content in largemouth bass

**Fig. 1:**
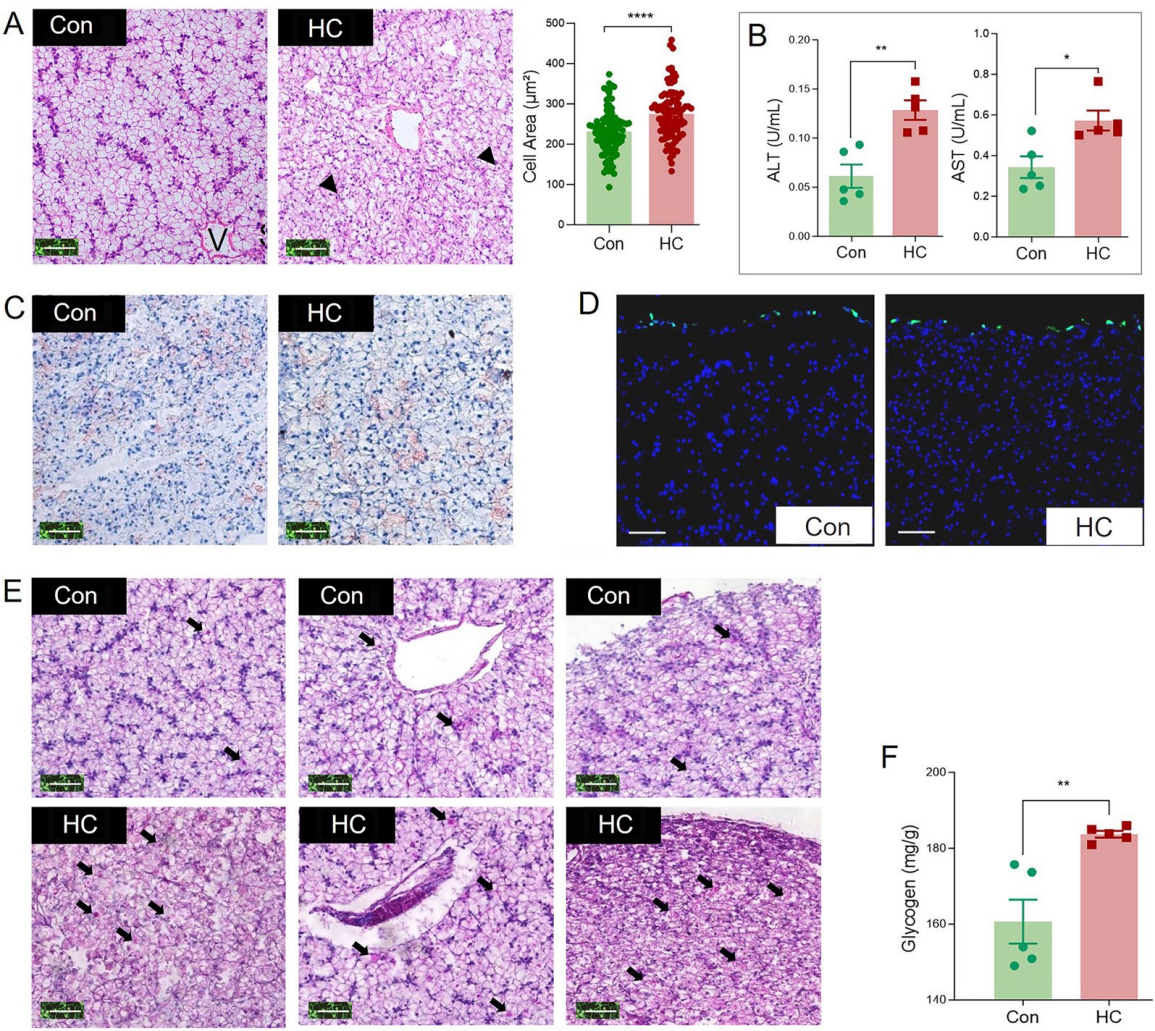
High carbohydrate diet induced liver injury with increased intrahepatic glycogen but not lipid content in largemouth bass. **A** H&E staining analysis of liver of largemouth bass in Con and HC groups. V stands for central vein, while S stands for hepatic blood sinuses. Black triangle marks irregular arrangement of nuclei. The white triangle marks where the cell membrane is broken. Scale: 50 μm. **B** The enzyme activities of ALT and AST in the serum of largemouth bass in Con and HC groups. Data are presented as means ± SEM (*n* = 5). **C** Oil red O staining in the liver of largemouth bass in Con and HC groups. Scale: 50 μm. **D** TUNEL staining in liver of largemouth bass and statistical analysis of TUNEL-positive cells in Con and HC groups. Scale: 50 μm. **E** PAS staining in the liver of largemouth bass in Con and HC groups. Black arrow: glycogen. Scale: 50 μm. **F** Glycogen content assay in the liver of largemouth bass in Con and HC groups. Data are presented as means ± SEM (*n* = 5). *: *P* < 0.05, **: *P* < 0.01

Hepatocytes, the main liver cell type in largemouth bass, are radially arranged around the central vein as cords forming cell plates, each of which separates several lacunae to form the vascular (sinusoids) and biliary (canaliculi) network. Moreover, sinusoids are present as the tubular form with narrow sinusoidal capillaries and irregularly shaped sinusoids appearing throughout the interstice between the hepatic plates. However, the HC group exhibited swollen hepatocytes with enlarged cell size, accompanied with the reduced radially arrangement of hepatocytes, damaged cell membrane, and peripherally located nuclei (Fig. 1A). Meanwhile, ALT activity in the serum of largemouth bass in the HC group was significantly higher than that in the Con group (*P* < 0.01, Fig. 1B). Similarly, largemouth bass in the HC group also exhibited significantly higher serum AST activity than the Con group (*P* < 0.05, Fig. 1B). Thus, the high carbohydrate diet significantly affected liver health in largemouth bass.

Oil red O staining indicated that lipid droplets in frozen liver slices of largemouth bass in both groups showed no significant difference (Fig. 1C). Biochemical analysis showed that hepatic contents of TG, T-CHO nor LDL-C were also not significantly affected by high carbohydrate diet (*P* > 0.05) (Supplementary Fig. S1A). Furthermore, no significant difference was detected in mRNA expression of genes related to lipogenesis (*acaca*, *fasn* and *srebf1*) nor lipolysis (*lpl* and *lipca)* (*P* > 0.05) (Supplementary Fig. S1B). Since liver steatosis always progresses to necrotizing inflammation followed by fibrosis and cirrhosis, TUNEL analysis was conducted. As shown in Fig. 1D, TUNEL-positive cells were mainly distributed in the margins of largemouth bass liver, and no significant difference was found between the two groups. Meanwhile, the activities of PCD-related enzymes including apoptosis-initiating caspase (caspase8 and caspase9), apoptosis-executing caspase (caspase3), and inflammatory caspase (caspase1) were not significantly affected by high carbohydrate diet (*P* > 0.05) (Supplementary Fig. S1C). Thus, high carbohydrate diet showed no significant effect on the lipid metabolism or lipid deposition in largemouth bass liver, induced neither liver apoptosis nor pyroptosis. Hepatic glycogen deposition in two groups was evaluated via PAS staining and biochemical analysis. As shown in Fig. 1E, accompanied with the affected liver structure, the HC group exhibited excessive glycogen accumulation in the liver after PAS staining. Biochemical analysis with the glycogen content detection kit indicated that liver glycogen content increased significantly (*P* < 0.01) from 161 mg/g in Con group to 184 mg/g in HC group (Fig. 1F). All these results suggest that the high carbohydrate diet significantly increased intrahepatic glycogen but not lipid content in largemouth bass.

### Metabolomics results confirmed the glycogenic hepatopathy rather than hepatic steatosis in largemouth bass induced by high carbohydrate diet

**Fig. 2:**
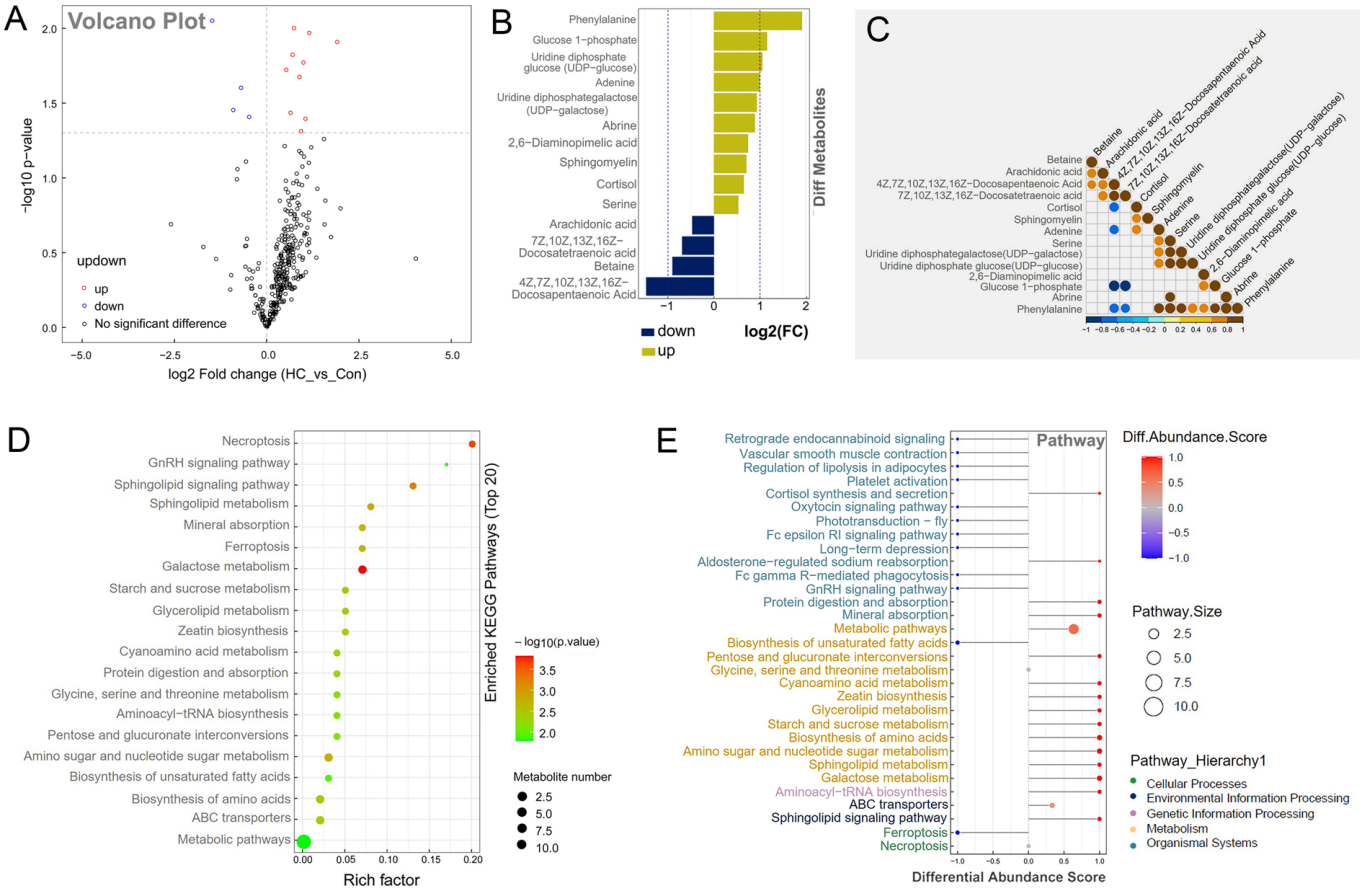
High carbohydrate diet affected hepatic metabolomics of largemouth bass. **A** Volcanic map of differential metabolites from the liver metabolome of largemouth bass between two groups. **B** Multiple analysis of significant differences in metabolites expression between two groups. **C** Correlation analysis of metabolites with significant differences between two groups. **D** KEGG enrichment pathway map of differential metabolites between two groups. **E** Differential abundance score maps of all differential metabolic pathways between two groups

High-throughput H650 targeted metabolomics analysis was performed to further verify the high carbohydrate-induced glycogenic hepatopathy in largemouth bass. Orthogonal partial least squares discriminant analysis (OPLS-DA) reflected the different pattern of metabolites distribution between two groups (Supplementary Fig. S2A). Volcanic map results showed that high carbohydrate diet resulted in significant differential levels of 14 metabolites, among which 10 metabolites significantly increased and 4 metabolites significantly decreased (Fig. 2A). The bar chart in Fig. 2B clearly showed the changes in the differential levels of metabolites detected. The significantly increased metabolites in HC group included phenylalanine, glucose-1-phosphate, UDP-glucose, adenine, UDP-galactose, abrine, 2, 6-diaminopimelic acid, sphingomyelin, cortisol, and serine. Most increased metabolites were related to carbohydrate metabolism, including glycogen synthesis precursors (UDP-galactose, UDP-glucose, glucose-1-phosphate). The significantly decreased metabolites in largemouth bass liver included betaine and three high unsaturated fatty acids (4Z,7Z,10Z,13Z,16Z-docosapentaenoic acid, 7Z,10Z,13Z,16Z-docosatetraenoic acid, and arachidonic acid). Correlation analysis on the differential metabolites showed that betaine was positively correlated with 4Z, 7Z,10Z,13Z,16Z-docosapentaenoic acid and arachidonic acid. Cortisol was positively correlated with sphingomyelin and adenine, and negatively correlated with 4Z,7Z,10Z,13Z, 16Z-docosapentaenoic acid. Phenylalanine is positively correlated with adenine, serine, glycogen synthesis precursors (UDP-galactose, UDP-glucose, and glucose-1-phosphate), 2, 6-diaminopimelic acid, and abrine. There was a negative correlation with 4Z,7Z,10Z,13Z,16Z-docosapentaenoic acid and 7Z,10Z,13Z,16Z-docosatetraenoic acid (Fig. 2C).

KEGG enrichment analysis showed that differential metabolites in two groups were mainly enriched in the metabolic pathway (Fig. 2D). As shown in Fig. 2E, the differential abundance scores of all differential metabolic pathways included cellular processes, environmental information processing, genetic information processing, metabolism, and organismal systems. In the sub-category of organismal systems, cortisol synthesis and secretion and aldosterone-regulation of sodium reabsorption pathway were up-regulated, whereas other pathways were down-regulated. In the sub-category of metabolism, only the biosynthesis of unsaturated fatty acids was down-regulated, whereas metabolic pathways and sugar metabolism were up-regulated, including starch and sucrose metabolism, pentose and glucuronate interconversions, and galactose metabolism. Thus, all these results further confirmed that high carbohydrate diet induced glycogenic hepatopathy rather than hepatic steatosis in largemouth bass.

### Integrated metabolomics in liver and serum identified betaine as a potential key metabolite in the glycogenic hepatopathy of largemouth bass

**Fig. 3:**
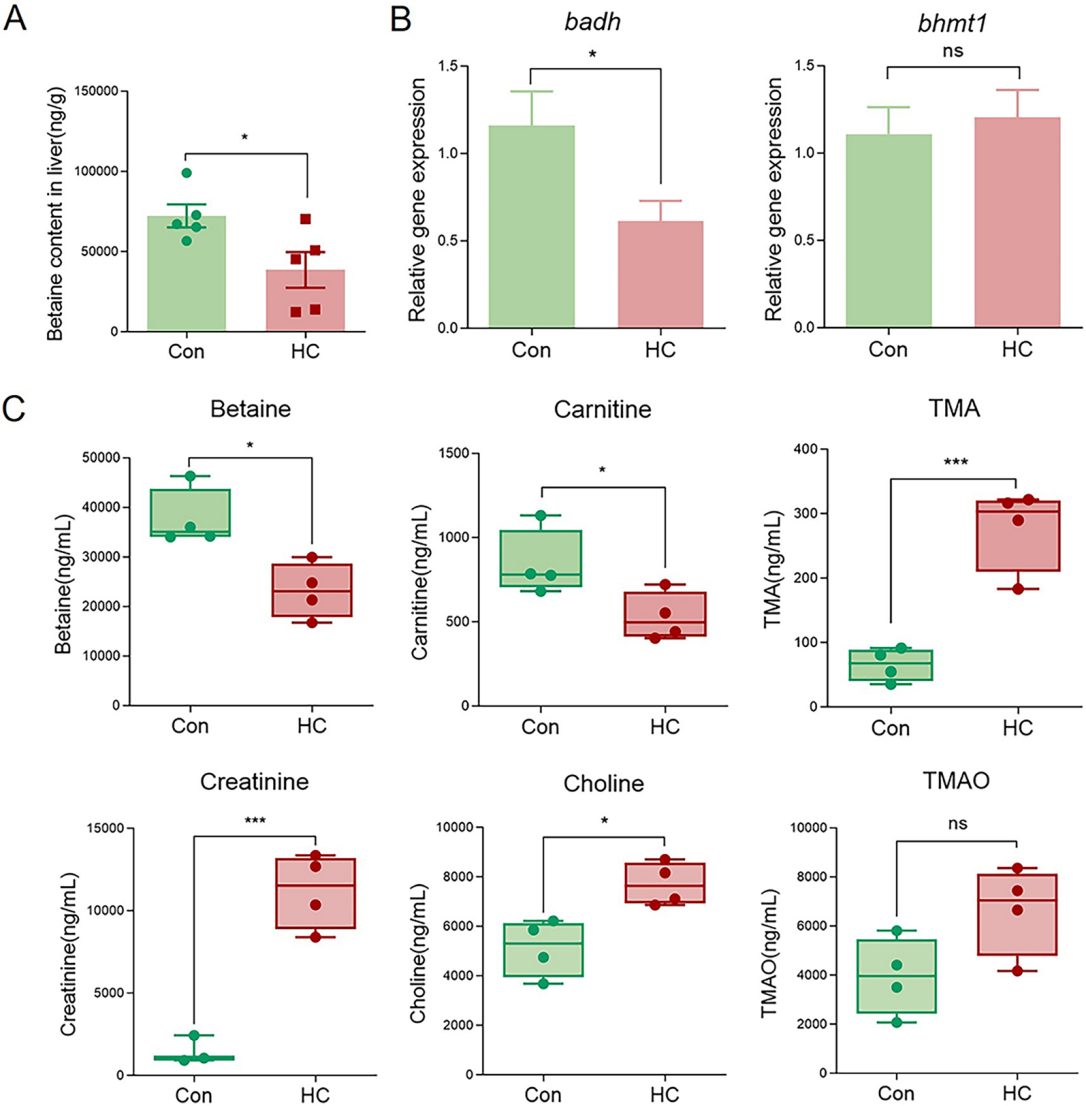
High carbohydrate diet significantly changed betaine metabolism in both liver and serum of largemouth bass. **A** The betaine content in the liver of largemouth bass in Con and HC groups. **B** The relative mRNA expression of genes related to betaine synthesis (*badh*) and decomposition (*bhmt1*) in liver of largemouth bass in Con and HC groups. **C** The contents of betaine-related metabolites (betaine, carnitine, TMA, creatinine, choline, TMAO) in serum of largemouth bass in Con and HC groups. Data are presented as means ± SEM (*n* = 5). *: *P* < 0.05, ***: *P* < 0.001, ns: *P* > 0.05

As mentioned in Fig. 2B, the decreased metabolites in the liver of the HC group were mainly concentrated in unsaturated fatty acids and betaine. Especially, betaine content was 38,560 ng/g in HC group, which was significantly lower than that in Con group (72,242 ng/g) (Fig. 3A). Meanwhile, mRNA expression of *badh,* which is involved in betaine synthesis, was significantly down-regulated in the HC group, whereas *bhmt1,* which was involved in betaine decomposition, showed no significant changes (Fig. 3B). Further targeted metabolomics evaluated serum contents of betaine-related metabolites including betaine, carnitine, creatinine, choline, TMA, and TMAO in two groups. As shown in Fig. 3C, high carbohydrate diet decreased serum contents of betaine and carnitine, increased serum contents of creatinine, choline, and TMA, whereas no significant differences were found in serum TMAO contents. Thus, integrated metabolomics results identified betaine as a potential key regulatory metabolite in glycogenic hepatopathy of largemouth bass.

Dietary betaine supplementation exhibited growth-promoting potential in largemouth bass during an eight-week feeding trial, as the differences in MFW, WGR, and SGR between HC and HC + Bet group increased with the extension of feeding time, although no significant changes were found in the test period (Supplementary Fig. S2B). Targeted metabolomics analysis indicated that serum contents of betaine and carnitine in HC + Bet group were significantly higher than that in HC group, whereas creatinine, choline, TMA, and TMAO contents showed no significant difference between two groups (*P* > 0.05) (Supplementary Fig. S3A). Metabolomics in liver showed that betaine content in liver significantly increased from 38,559 ng/g (HC group) to 86,083 ng/g (HC + Bet group) (*P* < 0.01) (Supplementary Fig. S3B). Further RT-qPCR analysis was conducted to detect the expression of betaine-related metabolic genes between two groups. As shown in Supplementary Fig. S3C, no significant change was found in *badh* expression, whereas betaine supplementation significantly increased the expression of *bhmt1* in bass liver (*P* < 0.05). All these results indicated that dietary betaine supplementation indeed affected betaine metabolism, resulting in the increased content of betaine and carnitine in largemouth bass.

### Dietary betaine supplementation reprogrammed liver metabolism and stress response in largemouth bass induced by high carbohydrate diet

**Fig. 4:**
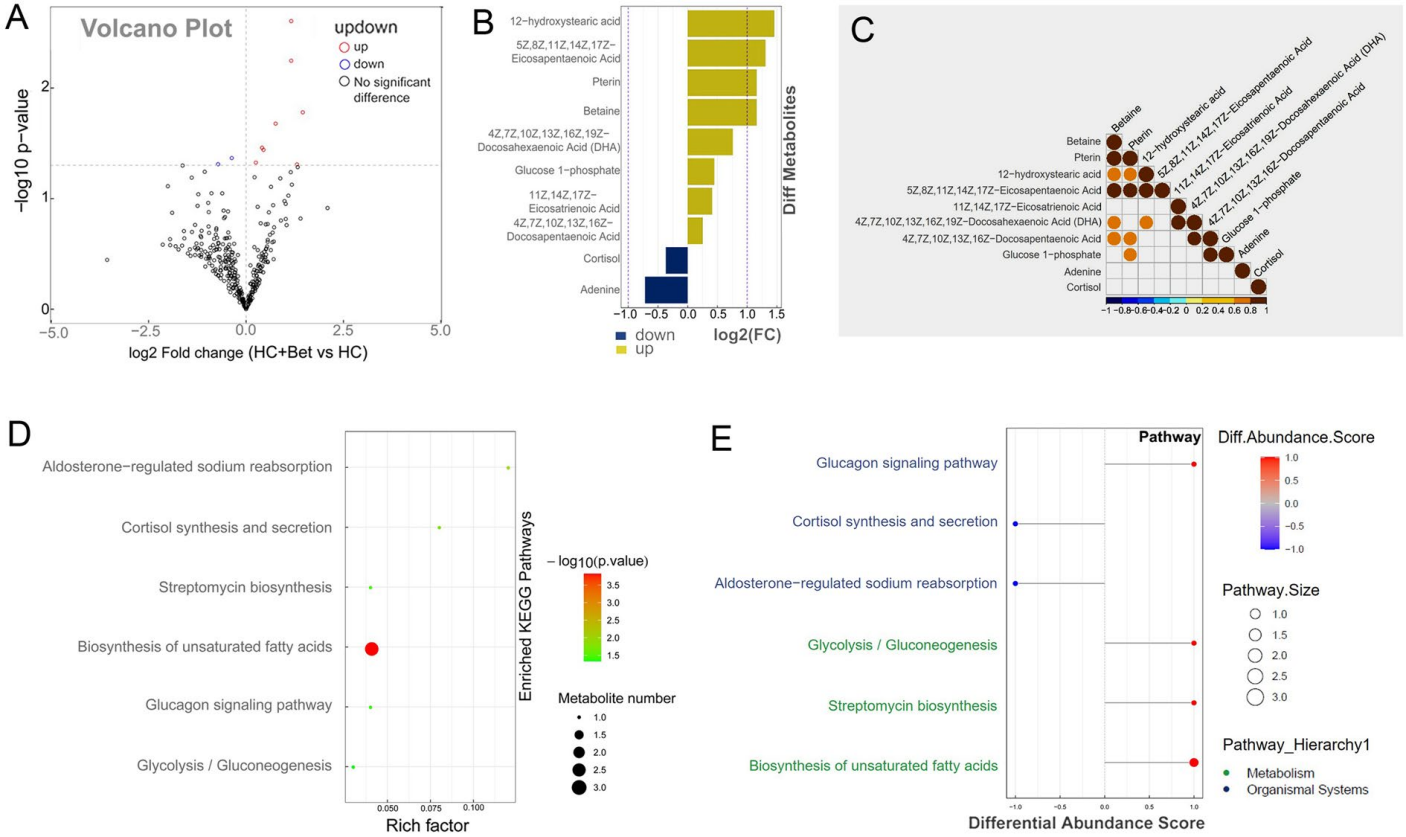
Dietary betaine supplementation significantly affected the hepatic metabolomics of largemouth bass. **A** Volcanic map of differential metabolites from the liver metabolome of largemouth bass. **B** Multiple analysis of significant differences in metabolite expression between two groups. **C** Correlation analysis of metabolites with significant differences between two groups. **D** KEGG enrichment pathway map of differential metabolites between two groups. **E** Differential abundance score maps of all differential metabolic pathways between two groups

H650 high-throughput targeted metabolomics was also conducted to analyze betaine effects on metabolic disorder of largemouth bass liver caused by high carbohydrate diet. OPLS-DA showed significant differences in metabolite expression between HC and HC + Bet group (Supplementary Fig. S4A). The volcanic map results showed that betaine supplementation resulted in significant changes in 10 metabolites, including 8 increased metabolites and 2 decreased metabolites (Fig. 4A). As shown in Fig. 4B, the bar chart clearly showed the changed ratio of significant differential metabolites detected. The 8 increased metabolites in the HC + Bet group included 12-hydroxystearic acid, 5Z,8Z,11Z,14Z,17Z-eicosapentaenoic acid, pterin, betaine, 4Z,7Z,10Z,13Z,16Z,19Z-docosahexaenoic acid, glucose-1-phosphate, 11Z,14Z,17Z-eicosatrienoic acid and 4Z,7Z,10Z,13Z,16Z-docosapentaenoic acid, whereas the 2 decreased metabolites included cortisol and adenine. Thus, dietary betaine may recover the decreased hepatic betaine and high unsaturated fatty acid content induced by high carbohydrate diet, and alleviated the stress response of largemouth bass induced by high carbohydrate diet. The correlation heat map further confirmed that there was a positive correlation between betaine and high unsaturated fatty acids (Fig. 4C).

KEGG enrichment analysis showed that differential metabolites were enriched in biosynthesis of unsaturated fatty acids (Fig. 4D). In order to further analyze the overall changes of KEGG metabolic pathways, the differential abundance scores of all differential metabolic pathways were exhibited in Fig. 4E, which were mainly enriched in metabolism and organismal systems. In the sub-category of organismal systems, glucagon signaling pathway was up-regulated, whereas cortisol synthesis and secretion, and aldosterone-regulated sodium reabsorption pathway were down-regulated. In the sub-category of metabolism, glycolysis and gluconeogenesis, streptomycin biosynthesis, and biosynthesis of unsaturated fatty acids were up-regulated. All the above metabolome results indicate that dietary betaine can relieve the liver metabolic disorder and stress response in largemouth bass induced by high carbohydrate diet.

### Dietary betaine supplementation alleviated glycogenic hepatopathy of largemouth bass induced by high carbohydrate diet

**Fig. 5:**
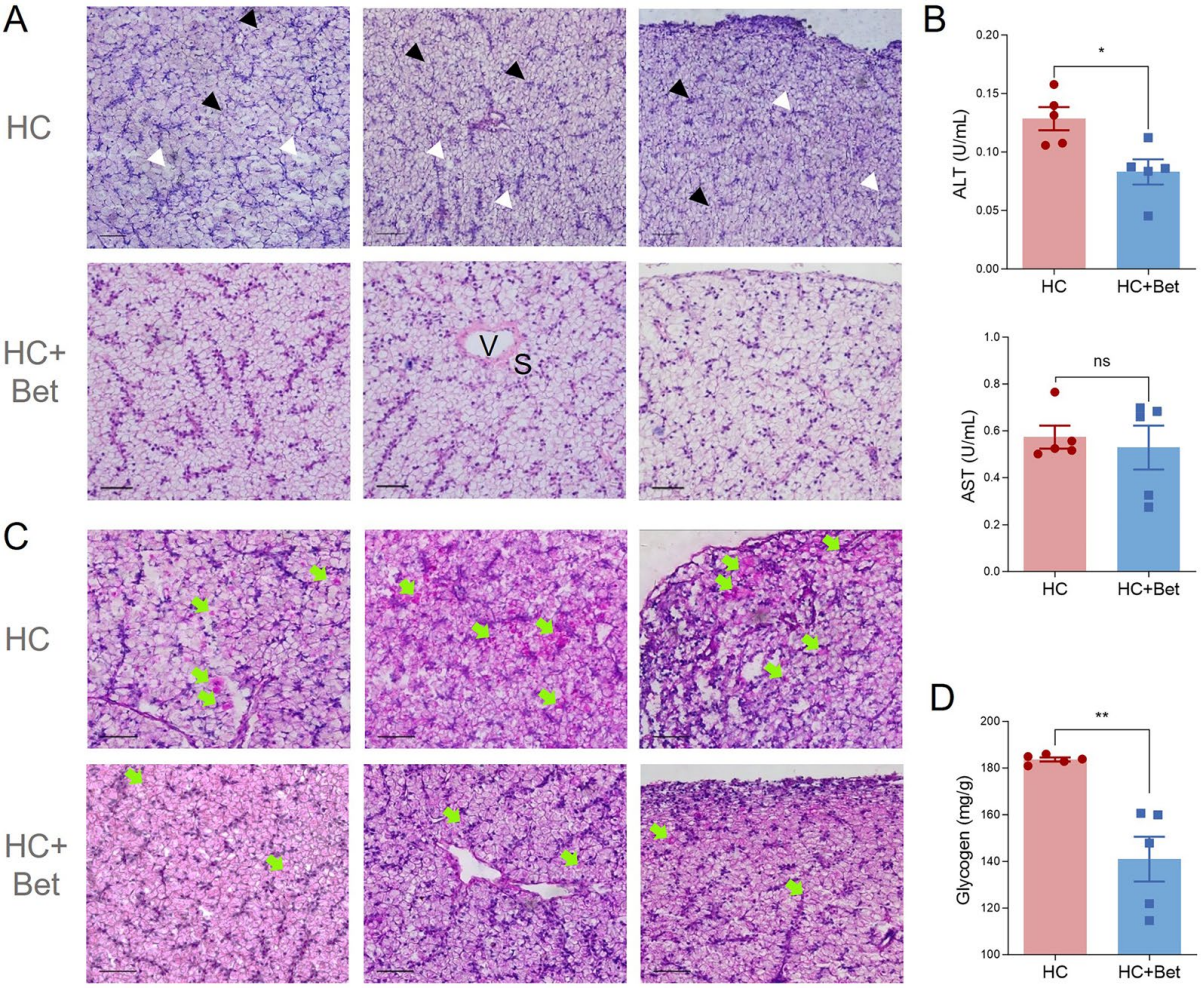
Dietary betaine supplementation significantly alleviated the high carbohydrate-induced liver histology, serum activities and hepatic glycogen deposition of largemouth bass. **A** H&E staining analysis of liver of largemouth bass in HC and HC + Bet groups. V stands for central vein, and S stands for hepatic blood sinuses. Black triangle marks irregular arrangement of nuclei. The white triangle marks where the cell membrane is broken. Scale: 50 μm. **B** Enzyme activities of serum ALT and AST in largemouth bass in HC and HC + Bet groups. Data are presented as means ± SEM (*n* = 5). *: *P* < 0.05, ns: *P* > 0.05. **C** PAS staining analysis of largemouth bass liver in HC and HC + Bet groups. Green arrow: glycogen. Scale: 50 μm. **D** Glycogen content assay of largemouth bass liver in HC and HC + Bet groups. Data are presented as means ± SEM (*n* = 5). **: *P* < 0.01

H&E staining indicated that the disordered cell arrangement, the destroyed structure of the hepatic sinuses, and the partially broken cell membrane induced by high carbohydrate diet were obviously improved by dietary betaine supplementation (Fig. 5A). In addition, the enzyme activity of ALT in serum of HC + Bet group was significantly lower than that in HC group (*P* < 0.05, Fig. 5B), whereas no significant change in the enzyme activity of AST was detected between the two groups (*P* > 0.05, Fig. 5B).

PAS staining with sections of largemouth bass liver indicated that, compared to the obvious accumulation of glycogen in the liver of largemouth bass in HC group, dietary betaine supplementation significantly decreased the accumulated hepatic glycogen (Fig. 5C). Biochemical analysis also showed the decreased glycogen content in largemouth bass liver after dietary betaine supplementation (Fig. 5D). All these results confirmed that dietary betaine supplementation significantly reduced the hepatic glycogen accumulation in largemouth bass induced by high carbohydrate diet. Conversely, no significant changes were found in related lipid indexes in HC + Bet group and HC group with oil red O staining, TG detection, T-CHO, and LDL-C detection (Supplementary Fig. S4B, D). However, the expression of the *acaca* gene in the lipid synthesis pathway was significantly down-regulated (*P* < 0.01), whereas the other lipid metabolism-related genes showed no significant changes (Supplementary Fig. S4C). No significant effect on TUNEL^+^ cell numbers or apoptosis-related enzyme activity was detected by betaine supplementation (Supplementary Fig. S4E, F).

### Dietary betaine supplementation increased largemouth bass resistance against ammonia stress

**Fig. 6:**
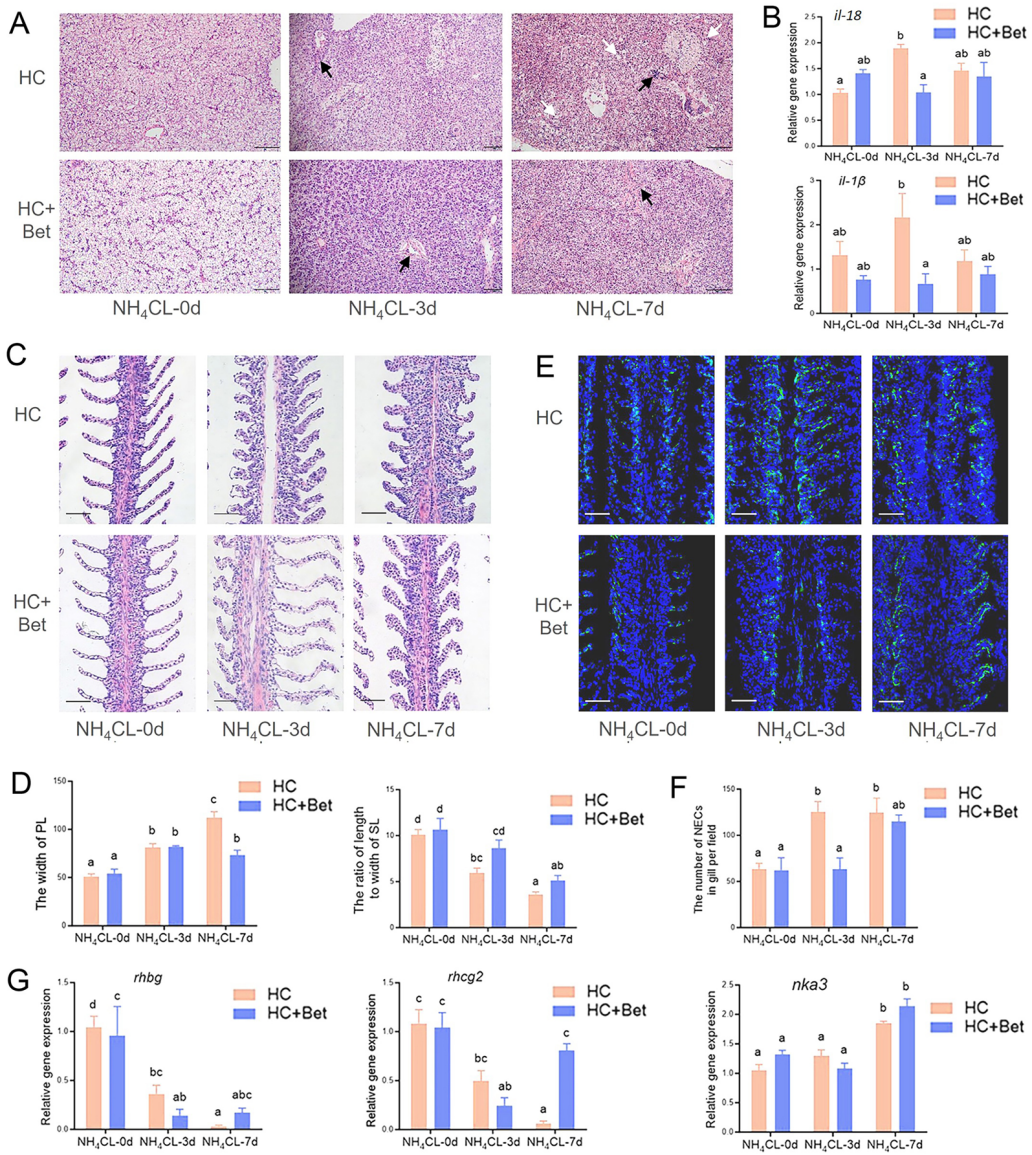
Dietary betaine supplementation significantly enhanced the resistance ability in liver and gill of largemouth bass against ammonia stress. **A** H&E staining assay of largemouth bass liver in HC and HC + Bet groups after ammonia stress for three and seven days, respectively. Black arrow: aggregation of inflammatory cells. White arrow: cavitation degeneration. Scale: 100 μm. **B** Relative mRNA expression levels of pro-inflammatory cytokines (*il18*, *il-1β*) in largemouth bass liver in HC and HC + Bet groups after ammonia stress for three and seven days. **C** H&E staining assay of largemouth bass gill in HC and HC + Bet groups after ammonia stress for 3 and 7 days. Scale: 50 μm. **D** Statistical analysis of gill-related parameters. **E** Immunofluorescence assay of NECs in largemouth bass gill in HC and HC + Bet groups after ammonia stress for three and seven days. Scale: 50 μm.** F** Statistical analysis of NECs numbers on gills. **G** Expression of genes involved in gas exchange and ion exchange (*rhbg*, *rhcg2*, *nka3*) in the gills of largemouth bass in HC and HC + Bet groups after ammonia stress for three and seven days. Data are presented as means ± SEM (*n* = 5)

Ammonia stress for three days induced obvious pathological changes in liver structure of largemouth bass, indicated by hepatocyte swelling, irregular cell arrangement, and inflammatory cell infiltration. With the extended stress period to seven days, HC group exhibited aggravated hepatocyte swelling and inflammatory cell infiltration, which was accompanied with the severe vacuolar degeneration, whereas no vacuolar degeneration was detected in HC + Bet group (Fig. 6A). Additionally, ammonia stress for three days up-regulated the expression of *il-18* and *il-1β* in HC group (Fig. 6B), whereas their expression at three days after ammonia stress in HC + Bet group was significantly lower than that in the HC group. Moreover, ammonia stress also induced pathological changes in gill tissues, indicated by the thickened primary gill filaments and the shortened secondary gill filaments, which all resulted in the decreased respiratory area (Fig. 6C). Statistical analysis indicated that the width of primary gill filaments in HC + Bet group after ammonia stress for seven days was significantly lower than that in HC group (*P* < 0.05), whereas the length-to-width ratio of secondary gill filaments in the HC + Bet group after ammonia stress for three and seven days was higher than that in the HC group (Fig. 6D). Immunofluorescence results indicated that the number of neuroepithelial cells (NECs) in largemouth bass gill significantly increased after ammonia stress. Moreover, the distribution of NECs also changed from the base of primary gill filaments to branchial lamella after ammonia nitrogen stress (Fig. 6E). Statistical analysis indicated that the number of NECs in HC + Bet group was significantly lower than HC group after ammonia stress for three days (Fig. 6F). Furthermore, ammonia nitrogen stress significantly decreased the mRNA expression of *rhbg* and *rhcg2*, which are related to gas exchange, in the gill of two groups (Fig. 6G). However, their expression in HC + Bet group was significantly higher than HC group at seven days after ammonia stress. Conversely, the expression of Na^+^/K^+^-ATPase (*nka3*), which is involved in ion exchange, significantly increased after seven days of ammonia stress, and there was no significant difference between the two groups (Fig. 6G).

## Discussion

### Liver biopsy and metabolomic analysis identified the HC-induced glycogenic hepatopathy in a primitive teleost model

The liver is a critical hub for numerous physiological processes including macronutrient metabolism, blood volume regulation, immune system support, endocrine control of growth signaling pathways, lipid and cholesterol homeostasis, and the breakdown of xenobiotic compounds (Trefts et al. 2017). Among macronutrients, carbohydrate not only serves as one of the main energy supplies but also exerts a comprehensive set of effects at the cellular, physiological, and ecological levels. In humans and other mammals, excessive intake of carbohydrates, especially free sugars, has been shown to be strongly associated with NAFLD and glycogenic hepatopathy, as a result of increased lipogenesis and glycogenesis. Unlike the systematic research on NAFLD, glycogenic hepatopathy is underdiagnosed, as glycogenic hepatopathy is difficult to separate from hepatic steatosis (Resnick et al. 2011). In humans, NAFLD may range from simple steatosis (non-alcoholic fatty liver (NAFL)), where there is fatty infiltration but no evidence of hepatocellular injury, to NASH where there is evidence of inflammation and ballooning, with or without fibrosis. Glycogenic hepatopathy is detected by several key features including (1) marked glycogen accumulation, leading to pale and swollen hepatocytes, (2) no or mild fatty change, (3) no or minimal inflammation, (4) no or minimal spotty lobular necrosis, and (5) intact architecture with no significant fibrosis (Torbenson et al. 2006). It is of great significance to explore the effect of glycogenic liver disease on a specific model in which glycogenic hepatopathy is developed independent of hepatic steatosis and to also establish the evaluation system for glycogenic hepatopathy.

Fish, as congenital diabetics, have a poor ability in carbohydrate utilization, and excessive intake of carbohydrates may lead to the disturbance of glucose and lipid metabolism, with the increased lipogenesis, which are detected in blunt bream (Prisingkorn et al. 2017). However, recent studies showed that largemouth bass with excessive starch intake develop glycogenic liver disease rather than hepatic steatosis (Romano et al. 2022), which makes it a potential model for the research of glycogenic hepatopathy. In our study, H&E staining indicated that the radially arranged hepatocytes around the central vein as cords forming cell plates disappeared in liver fed with high carbohydrate diet. Moreover, hepatocytes were swollen with enlarged cell size, along with the damaged cell membrane and peripherally located nuclei. This is similar to former studies in other teleosts (Prisingkorn et al. 2017). Moreover, enzyme activity experiments indicated that the activities of both ALT and AST in serum of largemouth bass were significantly increased by high carbohydrate diet, which is similar to earlier studies (Huang et al. 2022). Thus, diagnostic tools including both liver biopsies and liver enzyme indicated that high carbohydrate diet induced liver damage in the largemouth bass. To further define the sub-type of liver disease, several tools were further adopted to evaluate the lipid content in bass liver. Both oil red O staining and biochemical analysis results indicated that high carbohydrate diet showed no significant influence on the intrahepatic triglyceride (IHTG) level in largemouth bass, neither the intrahepatic T-CHO nor LDL-C contents. Additionally, the expression of genes involved in the lipogenesis pathway and lipolysis pathway showed no significant changes with a high carbohydrate diet. Considering NAFLD may develop into NASH with inflammation, ballooning and even fibrosis under severe conditions (Younossi 2019), TUNEL analysis was performed to detect the programmed cell death. Indeed, the number of apoptotic cells, mainly distributed in the margin liver, was also not significantly affected by high carbohydrate diet. Similar results were found on the activities of several caspases. All these data indicated that liver damage in largemouth bass induced by high carbohydrate diet was not due to hepatic steatosis with fatty infiltration or liver fibrosis. Conversely, liver biopsy via PAS staining and biochemical analysis with hepatic glycogen content indicated that high carbohydrate diet induced excessive glycogen accumulation in largemouth bass liver. Thus, glycogenic hepatopathy rather than hepatic steatosis might be the cause for liver damage induced by high carbohydrate diet. With the advancement of analytical techniques, metabolomics may identify and quantify multiple biomarkers simultaneously in a high-throughput manner. This is useful for the screening, diagnosis, and prognosis of diseases and the better understanding of the molecular pathways involved in the development and progression of diseases, which can facilitate the development of individualized prevention and treatment regimes (Wang et al. 2018). Thus, the high-throughput metabolomics was applied in identifying and quantifying multiple biomarkers simultaneously in the development and progression of glycogenic hepatopathy. H650 high-throughput targeted metabolomics analysis in largemouth bass liver identified 14 metabolites of significant difference between the Con and HC groups. High carbohydrate diet significantly increased the contents of two amino acids including phenylalanine and serine in largemouth bass liver. Indeed, the increased phenylalanine level may be closely related to its role serving as an insulin secreting agent, as phenylalanine has been reported to reduce the glucose-induced hyperglycemia (Nuttall et al. 2006). Accordingly, the serine synthesis pathway has been reported to be driven by high fructose in acute myeloid leukemia cells. Furthermore, among 10 significantly increased metabolites, three metabolites (UDP-galactose, UDP-glucose, glucose-1-phosphate) serve as glycogen synthesis precursors (Qiao et al. 2018). Further KEGG enrichment analysis showed that sugar metabolism in the sub-category of metabolism was up-regulated, including starch and sucrose metabolism, pentose and glucuronate interconversions, and galactose metabolism. Thus, high carbohydrate diet significantly affected sugar metabolism and promoted glycogen synthesis in largemouth bass liver. Other five significantly increased metabolites included adenine, abrine, 2, 6-diaminopimelic acid, sphingomyelin, and cortisol, which all showed no significant correlation with intrahepatic triglyceride. Moreover, the increased cortisol level, which functions as the main glucocorticoid in the stress response of animals, indicated that largemouth bass experienced stress with high carbohydrate diet. Also, KEGG enrichment analysis identified the up-regulated cortisol synthesis and secretion pathways in the sub-category of organismal systems. Conversely three high unsaturated fatty acids (4Z,7Z,10Z,13Z,16Z-docosapentaenoic acid, 7Z,10Z,13Z,16Z-docosatetraenoic acid, and arachidonic acid) were identified among four significantly decreased metabolites. Unsaturated fat acids have been reported to protect the liver via resisting oxidative stress (Liu et al. 2016) and other steps. Thus, metabolomics analysis further confirmed that high carbohydrate diet induced largemouth bass liver damage should be attributed to glycogenic hepatopathy rather than hepatic steatosis.

### The identification and validation of betaine roles in preventing fish glycogenic hepatopathy

High-throughput metabolomics not only functions in identifying and quantifying multiple biomarkers simultaneously in the development and progression of diseases but also facilitates the prevention and treatment of diseases. In the present study, besides three high unsaturated fatty acids, only betaine was the other significantly decreased metabolites in bass liver with a high carbohydrate diet. Analysis showed that betaine was positively correlated with 4Z,7Z,10Z,13Z,16Z-docosapentaenoic acid and arachidonic acid. Former studies have shown that the addition of high carbohydrate promotes the conversion of glucose to homocysteine, which increases the consumption of betaine, thus reducing the content of betaine in the liver (Xu et al. 2018). Accordingly, *badh* catalyzes betaine synthesis via the oxidation of betaine aldehyde in liver. Moreover, the down-regulated expression of *badh* also supported the decreased betaine content in largemouth bass liver with high carbohydrate diet. Further targeted metabolomics with bass serum indicated the contents of betaine and carnitine in serum with high carbohydrate diet also significantly decreased. Thus, integrated metabolomics results in liver and serum identified betaine as a potential key regulatory metabolite in glycogenic hepatopathy of largemouth bass, which may be prevented through the exogenous betaine supplementation.

The second experiment was designed to evaluate the preventing role of betaine in largemouth bass against HC-induced glycogenic hepatopathy. Previous studies have shown that betaine improves the growth performance of fish (Eissa et al. 2022). Here, although 1% betaine showed no significant influence on growth performance, the gap of MFW and WGR between the two groups was larger with the increased feeding periods. Thus, betaine supplementation had better growth-promoting potential for largemouth bass; significant differences could be detected with longer periods (Wang et al. 2022). More importantly, the disordered liver cell arrangement caused by high carbohydrate diet was significantly alleviated by betaine as determined in liver biopsies via H&E staining. Such histological structure was accompanied by reduced serum ALT levels. This is similar to previous studies in rats, namely, that betaine treatment reduced serum ALT and AST levels (Hasanzadeh-Moghadam et al. 2018). To elucidate the hepatoprotective role of betaine, the present study examined the changes of glycogen in the liver of largemouth bass by PAS staining and biochemical analysis. Results showed that betaine supplementation significantly reduced the over-accumulated glycogen in largemouth bass liver induced by high carbohydrate diet. In order to illustrate the regulatory mechanism of dietary betaine supplementation, further targeted metabolomics was conducted. KEGG pathway enrichment analysis indicated that dietary betaine supplementation up-regulated the glucagon signaling pathway, unsaturated fat acid biosynthesis, glycolysis, and gluconeogenesis. It is noteworthy that glucagon is a counter-regulatory hormone that promotes hepatic glucose production (Kulina and Rayfield 2016). Such results may suggest that dietary betaine supplementation may upregulate the glucagon signaling pathway and enhance glucose utilization by increasing glycolysis, therefore ameliorating the disorders of glucose metabolism in largemouth bass induced by high dietary carbohydrate. Therefore, for the first time our study illustrated the alleviating roles of betaine on glycogenic hepatopathy in animals, including fish. Moreover, dietary betaine supplementation significantly increased serum betaine and carnitine content in largemouth bass fed with high carbohydrate diet. This is similar to previous studies in mammals and *Megalobrama amblycephala* insofar as exogenous addition of betaine increased the content of betaine and carnitine (Atkinson et al. 2008). In fact, betaine content in the liver also significantly increased with dietary betaine supplementation, which was accompanied with the significantly increased expression of *bhmt* gene. This may catalyze the reaction of betaine and Hcy for the synthesis of methionine. Thus, exogenous betaine supplementation results in both the increased deposition of betaine in liver and the increased betaine catabolism, which is consistent with previous research (Qian and Song 2011). Moreover, exogenous betaine supplementation also significantly increased the polyunsaturated fatty acid level in serum, which is in accordance with the positive correlation between betaine and 4Z,7Z,10Z,13Z,16Z-docosapentaenoic acid and arachidonic acid. Such results are also similar with previous studies in lambs and broiler chickens (Dong et al. 2020). Additionally, the contents of cortisol and adenine were significantly decreased with betaine supplementation. Thus, the decreased cortisol level suggested that betaine alleviates the stress response of largemouth bass induced by high carbohydrate diet.

### Dietary betaine supplementation enhanced fish resistance against ammonia challenge

A further ammonia stress experiment was conducted to explore the beneficial roles of betaine supplementation on fish stress resistance capacity. In consistent with previous reports (Zou et al. 2023), ammonia nitrogen stress for three days induced significant histopathological changes in liver, including swelling hepatocytes, irregular cell arrangement, and inflammatory cell infiltration. With the prolonged ammonia stress period to seven days, vacuolar degeneration occurred in the high carbohydrate group, whereas no vacuolar degeneration was detected with betaine supplementation. These results indicated that betaine protected the liver structure of largemouth bass during ammonia nitrogen stress, which is consistent with the conclusion that betaine can protect liver health in previous studies (Day and Kempson 2016). In addition to the histological changes, the RNA expression levels of *il-18* and *il-1β* in liver were significantly up-regulated after ammonia stress, which indicated the strong inflammatory response. However, dietary betaine supplementation significantly alleviated the up-regulated expression of these inflammatory factors. This is similar to previous studies, which reported that betaine could alleviate liver disease by inhibiting inflammatory reaction (Wang et al. 2021). A further confirmatory experiment with the beneficial roles of betaine supplementation on fish stress resistance ability was conducted in gills. Thus, ammonia stress for three days induced the thickened primary gill filaments and the reduced aspect ratio of the secondary gill filaments. However, the beneficial roles of betaine were detected with ammonia stress for seven days, as betaine supplementation significantly reduced the primary gill filament thickening of largemouth bass caused by ammonia nitrogen stress. In fact, fish gill filament is covered by two layers of respiratory epithelium, supported by a middle columnar cell as a blood convection channel to facilitate effective gas exchange between blood and water. NECs are believed to be chemical receptors for ammonia and mediators of hyperventilation reactions associated with high environmental ammonia (Perry and Tzaneva 2016). In the present study, ammonia stress induced the significantly increased number of NECs in bass gills, whereas dietary betaine supplementation significantly alleviated the increased number of NECs. This indicates the beneficial role of dietary betaine supplementation on the hyperventilation reaction of largemouth bass induced by high ammonia environment. Additionally, Rh family proteins related to gas exchange is responsible for the important respiratory role of gill in fish. In our study, ammonia stress resulted in the decreased mRNA expression of *rhbg* and *rhcg2* in the gills. As ammonia can enter fish bodies through Rh channels under high environmental ammonia conditions, the down-regulated expression of Rh family proteins may be a barrier mechanism formed to reduce the entry of external ammonia. However, the mRNA expression of *rhcg2* in HC + Bet group returned to the basic level after ammonia stress for seven days, indicating that dietary betaine may help largemouth bass adapt to the high ammonia environment. This assumption is reasonable as betaine functions as an important osmoregulator and an alternative methyl donor for homocysteine (Ueland 2011).

## Supplementary Information

Below is the link to the electronic supplementary material.

Supplementary file1 (DOCX 285 KB)

## Data Availability

Data are available from the corresponding author upon reasonable request.
