# Supplementary material for: Glycogenic hepatopathy in a primitive teleost fish model: the inductive effect of high carbohydrate diet and the alleviating role of betaine
Source: Mar Life Sci Technol. 2025 May 12;8(1):129–43. doi: 10.1007/s42995-025-00301-0 (PMC12953848; doi:10.1007/s42995-025-00301-0)
Supplement: Supplementary file 1 — Supplementary file1 (DOCX 285 KB) [file 42995_2025_301_MOESM1_ESM.docx]

**Supplementary Table S1** All primers used in the experiment

| Primers | Primer sequence （5'- 3'） | Gene ID |
| --- | --- | --- |
| *18S* | F: GCAAAGCTGAAACTTAAAGGAATTG | XR_005447146 |
|  | R: TCCCGTGTTGAGTCAAATTAAGC |  |
| *acaca* | F: TCCCCATCATCACTGGACAC | XM_038709737 |
|  | R: AGGCTGCAAATACGGTGGAG |  |
| *fasn* | F: CGGGTTGACCTGGGAAGAAT | XM_038735140 |
|  | R: ACTAATCGCTTCCTGCGGAC |  |
| *srebf1* | F: CCTGCATTTTACCTCCCGTG | XM_038699585 |
|  | R: TAGGTGTGCTGCGAATAGCC |  |
| *lpl* | F: CATGGCTGGACGGTAACAGG | XM_038715978 |
|  | R: GTCAGCCAGTCCACAACGAT |  |
| *lipca* | F: CAGTATGGGCTCCTCGGTTTT | XM_038701258 |
|  | R: TTGTCACTGCACCTGTAGGC |  |
| *badh* | F: AGAGGCTTGGAAACGTTGTA | XM_038718517 |
|  | R: CCGAGGACAGGCATCTAACC |  |
| *bhmt1* | F: GGCAACGGGAAAGAAGGGAAT | XM_038703758 |
|  | R: TCTGCATAACGTTGGACCCG |  |
| *il-18* | F: TTTTTCCCCAGGTCTTCCTGATGG | XM_038692744 |
|  | R: GTGTCCTCATCCCAATCTGTTTCA |  |
| *il-1β* | F: TCAGCCACGGAGGAAAAAGAC | XM_038733429 |
|  | R: ACCTACATCAGGTGAGGTCTCTAA |  |
| *cxcr3* | F: CGACGCTACCGAAACTCCCA | XM_038710986 |
|  | R: AGCCAAGCCGAACACCCACT |  |
| *rhbg* | F: CCTCATGGTGACCCGAATCC | (Egnew et al 2019) |
|  | R:  TATGTGGACAGAGTGCAGGC |  |
| *rhcg2* | F: TGGCTACCTGTTTGTCACGC | (Egnew et al 2019) |
|  | R: TATAAAGCCGCCGAGCATCC |  |
| *nka3* | F: TGGTGATTCGTCCTCACACAG | XM_038693304 |

**Supplementary Figures**


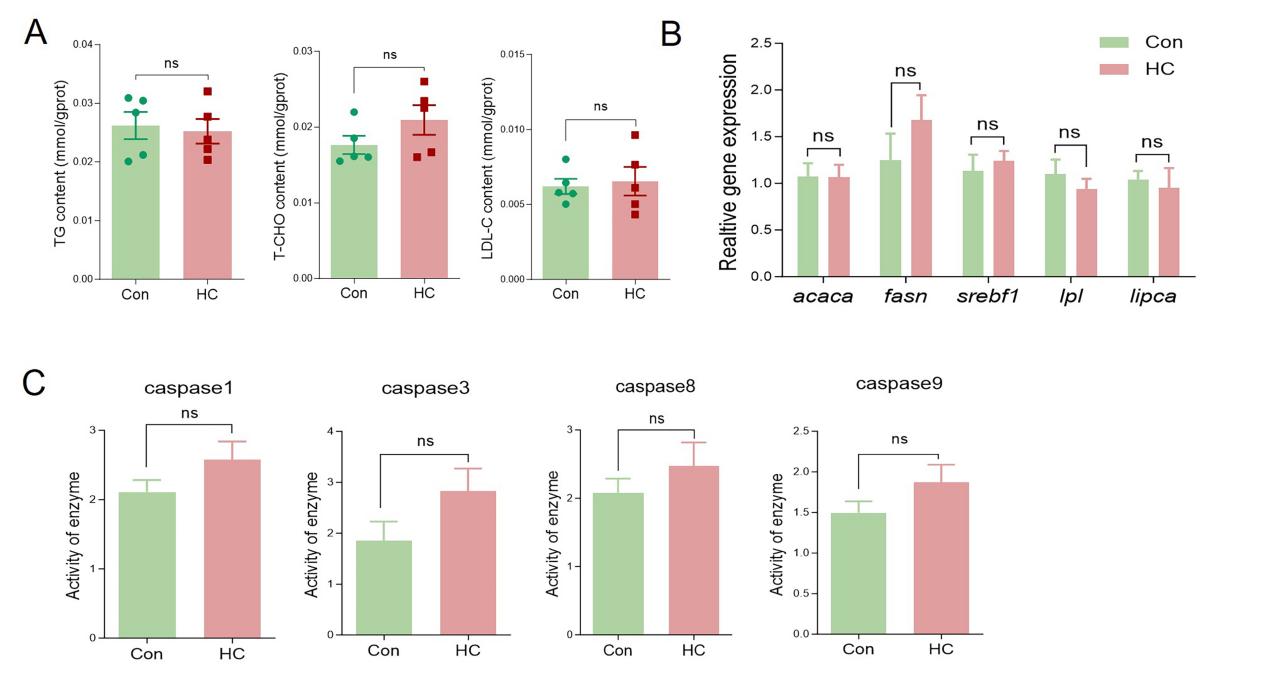


**Supplementary Fig. S1 High carbohydrate diet did not affect the contents of lipids, nor the RNA expression of genes involved in lipid metabolism (*acaca, fasn, srebf1, lpl, lipca*) or caspases (caspase-1,3,8,9) in largemouth bass liver.** ns: *P*>0.05.

**
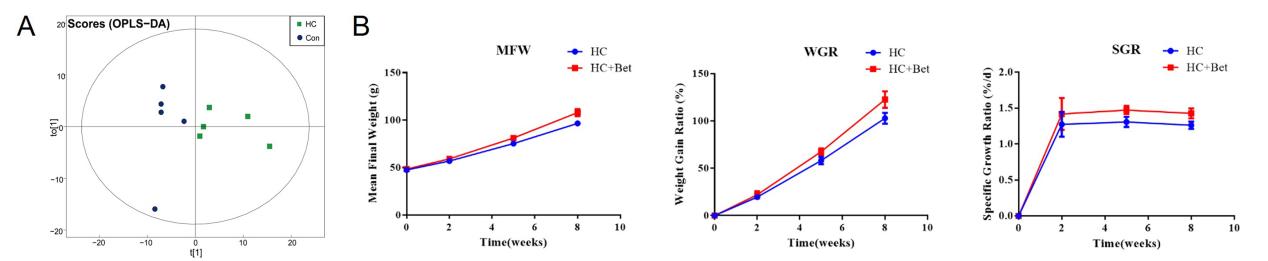
**

**Supplementary Fig. S2 Effect of dietary betaine supplementation on fish growth performance and OPLS-DA of hepatic metabolites in largemouth bass.** (A) OPLS-DA of liver metabolites in largemouth bass in Con and HC groups. (B) Growth parameters of largemouth bass (MFW: Mean final weight, WGR: weight gain rate, SGR：specific growth rate) were calculated. Data was presented as means ± SEM (n = 5).


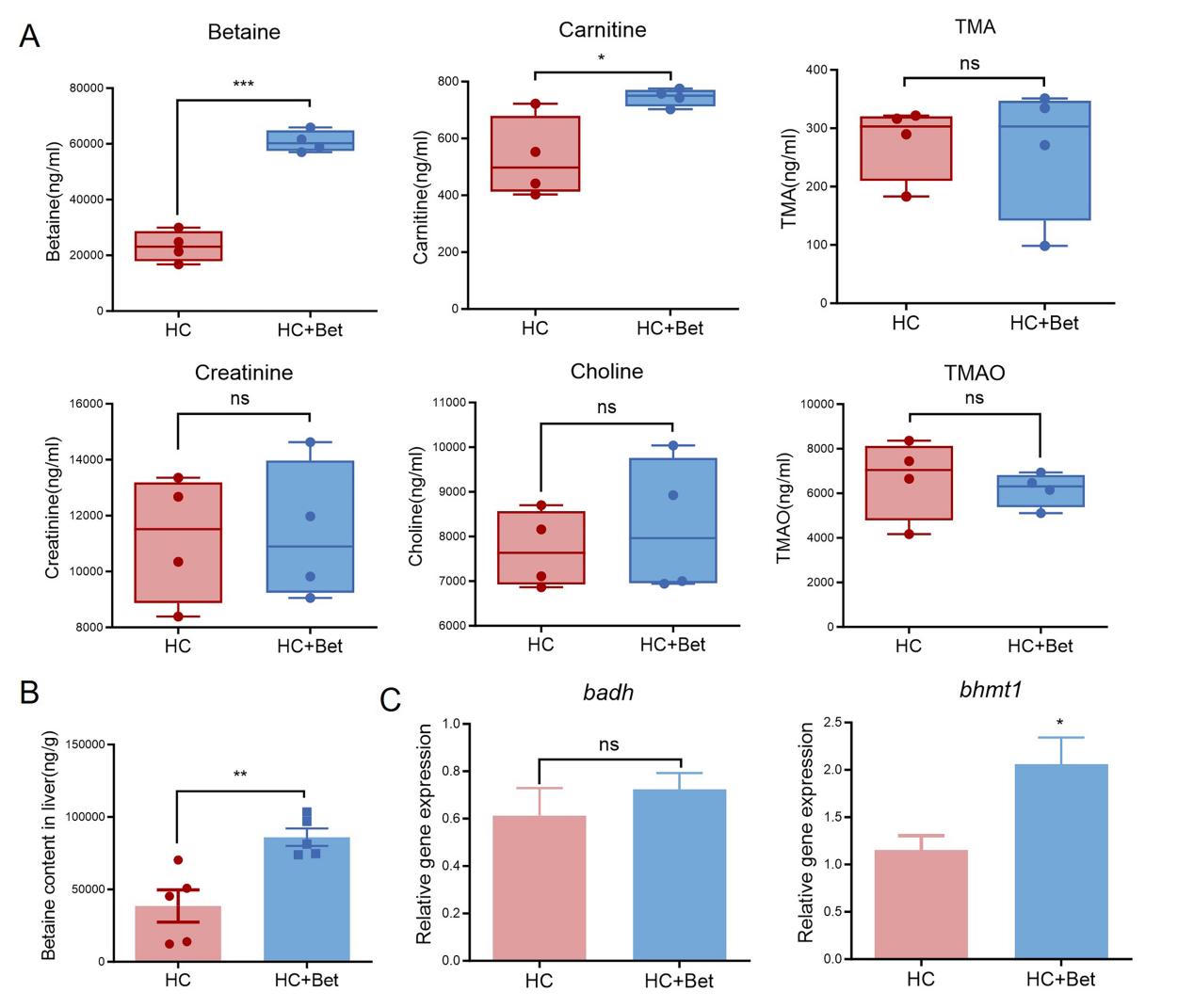


**Supplementary Fig. S3** **Dietary betaine supplementation reprogrammed the betaine-related metabolism in largemouth bass**. (A) The contents of betaine related metabolites (betaine, carnitine, TMA, creatinine, choline, TMAO) in serum of largemouth bass in HC and HC+Bet groups. (B) The contents of betaine in liver of largemouth bass in HC and HC+Bet groups. (C) The relative mRNA expression of genes involved in betaine synthesis (*badh*) and decomposition (*bhmt1*) in liver of largemouth bass in HC and HC+Bet groups. Data was presented as means ± SEM (n = 5), *: *P*<0.05，**: *P*<0.01，*** : *P*<0.001，ns: *P*>0.05.


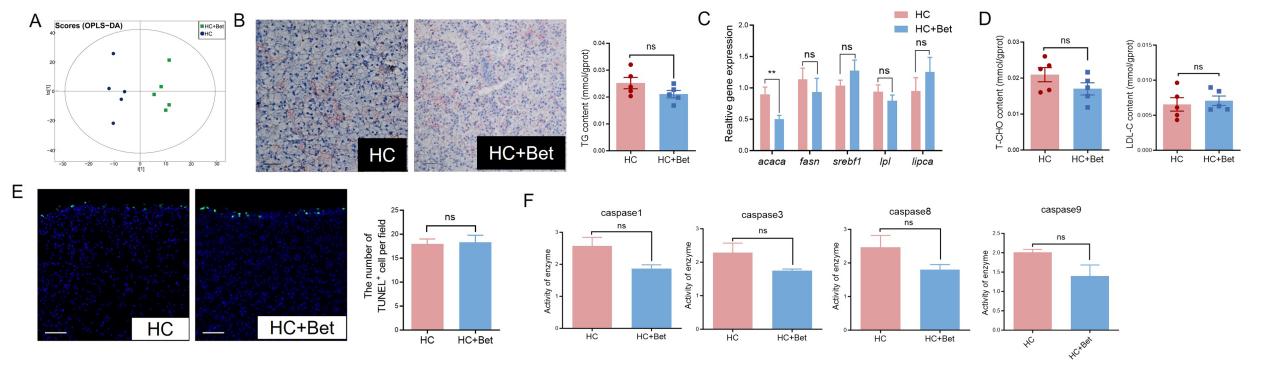


**Supplementary Fig. S4 Effect of dietary betaine supplementation on OPLS-DA of liver metabolites, lipid metabolism and programmed cell death in largemouth bass.** (A) OPLS-DA of liver metabolites in largemouth bass in HC and HC+Bet groups. (B) Oil Red O staining and TG content detection in largemouth bass liver in HC and HC+Bet groups. Scale: 50 μm, Data was presented as means ± SEM (n = 5). (C) Relative mRNA expression of genes related to lipid metabolism (*acaca, fasn, srebpf1,lpl* and *lipca*) in largemouth bass liver in HC and HC+Bet groups. Data was presented as means ± SEM (n = 5). (D) The contents of T-CHO and LDL-C in largemouth bass liver in HC and HC+Bet groups. Data was presented as means ± SEM (n = 5), ns: *P*>0.05, **: *P*<0.01. (E) TUNEL staining in largemouth bass liver and and statistical analysis of TUNEL-positive cells number in HC and HC+Bet groups. Scale: 50 μm. (F) Activity assay of enzymes related to programmed cell death in largemouth bass liver in HC and HC+Bet groups. Data was presented as means ± SEM (n = 5), ns: *P*>0.05.
